# Alterations of the Gut Microbiome and Metabolome in Patients With Proliferative Diabetic Retinopathy

**DOI:** 10.3389/fmicb.2021.667632

**Published:** 2021-09-08

**Authors:** Panpan Ye, Xueyou Zhang, Yufeng Xu, Jia Xu, Xiaoxiao Song, Ke Yao

**Affiliations:** ^1^Eye Center, Second Affiliated Hospital, School of Medicine, Zhejiang University, Hangzhou, China; ^2^Eye Hospital, Zhejiang University, Hangzhou, China; ^3^Department of Surgery, First Affiliated Hospital, School of Medicine, Zhejiang University, Hangzhou, China; ^4^Department of Endocrinology, Second Affiliated Hospital, School of Medicine, Zhejiang University, Hangzhou, China

**Keywords:** diabetic retinopathy, microbiome, metabolome, cross-omics analysis, diagnostic targets

## Abstract

Diabetic retinopathy (DR) has been reported to associate with gut microbiota alterations in murine models and thus “gut-retina-axis” has been proposed. However, the role of gut microbiome and the associated metabolism in DR patients still need to be elucidated. In this study, we collected fecal samples from 45 patients with proliferative diabetic retinopathy (PDR) and 90 matched diabetic patients (1:2 according to age, sex, and duration of diabetes) without DR (NDR) and performed 16S rRNA gene sequencing and untargeted metabolomics. We observed significantly lower bacterial diversity in the PDR group than that in the NDR group. Differential gut bacterial composition was also found, with significant depletion of 22 families (e.g., *Coriobacteriaceae*, *Veillonellaceae*, and *Streptococcaceae*) and enrichment of two families (*Burkholderiaceae* and *Burkholderiales_unclassified*) in the PDR group as compared with the NDR group. There were significantly different fecal metabolic features, which were enriched in metabolic pathways such as arachidonic acid and microbial metabolism, between the two groups. Among 36 coabundance metabolite clusters, 11 were positively/negatively contributed to PDR using logistic regression analysis. Fifteen gut microbial families were significantly correlated with the 11 metabolite clusters. Furthermore, a fecal metabolite-based classifier was constructed to distinguish PDR patients from NDR patients accurately. In conclusion, PDR is associated with reduced diversity and altered composition of gut microbiota and specific microbe-metabolite interplay. Our findings help to better understand the disease pathogenesis and provide novel diagnostic and therapeutic targets for PDR.

## Introduction

Diabetes mellitus is growing fast and estimated to affect 10.2% (578 million) of the global population in 2030 (Saeedi et al., 2019). Diabetic retinopathy (DR) is its most common microvascular complication and a major cause of the worldwide vision impairment (Duh et al., 2017). Proliferative diabetic retinopathy (PDR) is the advanced stage of DR and the leading cause of blindness. The pathological hallmarkers of PDR include capillary occlusion and neovascularization (Duh et al., 2017). Accordingly, intravitreous injection of anti-VEGF drugs, laser, and vitrectomy have been developed to treat PDR. Although these treatments seem to be effective, some of the treated eyes still deteriorate and lose vision finally (Duh et al., 2017), necessitating the development of novel therapeutic approaches.

Recent progresses in basic science greatly improve our knowledge about how diabetes impacts retina such as the proposed “gut-retina axis.” Gut microbiota is the most densely populated microbial ecosystem in human body and is integral to the maintenance of human health. Alterations in the gut microbiota have been implicated in the onset and development of various human diseases including diabetes (Wu et al., 2020) and ocular disorders (Floyd and Grant, 2020). Previous studies in murine models have demonstrated that type 2 diabetes-altered gut microbiota is associated with the exacerbation of DR, indicating a possible role of gut-retina axis (Floyd and Grant, 2020). Targeting the gut microbiota and their-associated metabolites such as tauroursodeoxycholate (TUDCA) *via* diet or probiotics might be a new therapeutic strategy (Floyd and Grant, 2020). However, the specific gut microbiota composition and the related metabolic profiling in DR, particularly the highly blindness-causing PDR, is still unknown in human. We herein conducted the pioneering human study to identify the pathological mechanism of PDR from a deep understanding of gut-retina axis and to seek the novel predictive and therapeutic targets.

## Materials and Methods

### Study Cohort and Samples

We recruited patients with type 2 diabetes in Eye Center, the Second Affiliated Hospital, Zhejiang University School of Medicine between January 2020 and July 2020. This study was approved by the ethical committee of the Second Affiliated Hospital, Zhejiang University School of Medicine (approval number 2020776) and conformed to the ethical guidelines of the 1975 Declaration of Helsinki. Written informed consents were obtained from all subjects. The inclusion criteria were all adult patients with age > 18 years, Han population, a history of type 2 diabetes, ECOG score 0–1, receiving visual acuity, dilated fundoscopy, optical coherence tomography (OCT), and fundus fluorescein angiography (FFA). The exclusion criteria were as follows: sepsis in need of antibiotics within 12 weeks prior to enrollment, specifically infection such as hepatitis virus or tuberculosis, cancers, severe systemic diseases, and hospitalized, unhealthy lifestyle such as alcohol abuse and smoking. The patients with the presentation of retinal neovascularization in FFA were diagnosed as PDR. During the study period, a total of 45 enrolled patients were diagnosed as PDR and received vitrectomy (PDR group, *n* = 45). Another 90 matched patients (1:2 according to age, sex, and duration of diabetes) without DR were included using propensity score (NDR group, *n* = 90). Stool samples were obtained and immediately frozen in liquid nitrogen before storage at −80°C. Clinical data including biochemistry were acquired and analyzed.

### DNA Extraction and 16S rRNA Gene Sequencing

DNA was extracted using a kit (TIANGEN Biotech, Beijing, China). The V3–V4 region of the bacterial 16S rRNA gene was amplified and sequenced using an Illumina HiSeq2500 platform (Illumina, San Diego, CA, United States) as we described previously (Lu et al., 2019). Quality-filtered sequences were clustered into operational taxonomic units (OTUs) and displayed using R software (version 2.15.3). Bacterial richness and diversity analyses were calculated. Principal component analysis (PCA) using weighted and UniFrac distance metrics were conducted. Wilcoxon rank-sum test was performed for comparison between the two groups. Linear discriminant analysis (LDA) effect size (LEfSe) method was used for distinguishing taxonomic types. Phylogenetic investigation of communities by reconstruction of unobserved states (PICRUSt) analysis was performed to predict functional composition profiles.

### Liquid Chromatography-Mass Spectrometry-Based Metabolomics Analysis

Fecal samples were used for liquid chromatography-mass spectrometry-based metabolomics (UPLC-MS) analysis as we described previously (Zhang et al., 2020). A portion of 100 mg sample was weighted precisely and mixed with 1 ml of chilled methanol/deionized water (4:1, *v*:*v*) and homogenized at 5,000 rpm for 5 min by a Precellys 24 (Bertin Technologies, Paris, France) with 200 mg glass beads (Sigma-Aldrich, St Louis, MO, United States). The homogenate solution was then bathe in ice for 20 min and centrifuged at 15,000 rpm for 10 min. Finally, the supernatant was lyophilized and reconstructed with 200 μl methanol/water (4:1, *v*:*v*) for UPLC-Q-TOF/MS analysis. Quality control (QC) samples were preprocessed in the same method for data quality assessment. A Waters Q-TOF Premier mass spectrometer (Waters, Manchester, United Kingdom) was used to perform the mass spectrometry in both positive and negative electrospray ionization (ESI) mode. The raw data were analyzed with Compound Discoverer 3.0 (CD 3.0, Thermo Fisher Scientific). Peaks were matched with the database of mzCloud^1^ and ChemSpider^2^. MetaboAnalyst 4.0 and SIMCA software were used for functional analysis (Zhang et al., 2020). Batch effect was removed with quantile normlization. The ESI+ and ESI− mode features were then concatenated for the downstream analysis. Samples that failed to exceed 50% meaning values would be excluded. Zero values were replaced by the half of the minimum positive value on a per-sample basis. Feature filtering based on median absolute deviation were performed to remove noise. Individual features were log-transformed to variance stabilize the data. During correlation calculation, if multiple metabolomic features/compounds matched the same MS2 metabolites, the feature/compound with the highest absolute correlation would be selected.

### Unsupervised Clustering of Coabundant Metabolites

Clusters of coabundant metabolites were identified using the R package WGCNA. Top 75% median absolute deviation of identified MS1/MS2 features were filtered to cluster a signed and weighted metabolite coabundance correlation network. The correlation method in all functions was biweight midcorrelation (bicor) (maxPOutliers = 0.05) because of its robustness (Langfelder and Horvath, 2012). The power value of our scale-free topology was 3.

### Cross-Omics Correlation Network Analysis

Spearman correlations between the gut microbiota and metabolite modules were performed using R 4.0.2. We also performed biweight midcorrelation (Langfelder and Horvath, 2012) to calculate correlation between metabolite modules and clinical phenotype. Adjusted *p*-values (FDR) were controlled by the Benjamini–Hochberg method. Logistic regression was performed using stats R package. Simulated Residuals test was performed using DHARMa R package. Multicollinearity test was performed using car R package.

### Support-Vector Machine Classification

We performed support-vector machines (SVMs) with Radial asis Function Kernel classification using the implementation of this method in caret R package. We considered SVM classifiers for predicting NDR/PDR status. SVM was trained on 70% of the samples and tested on the remaining 30%. Features were not selected in any way before training. Data were scaled and centered before training, and cross-validated (fivefold and repeated 100 times) for resampling. Parameter used for the model were sigma = 0.0002563017 and C = 0.25. The performance of models was measured by area under curve (AUC), which was calculated using pROC R package.

### Statistical Analysis

The significance was compared using Student’s *t*-test, ANOVA, or the Kruskal–Wallis test. Other statistical approach in dataset bioinformatics analyses has been described above. Data were analyzed using GraphPad Prism software version 6.1 (GraphPad software, Inc.), R version 4.0.2 (R foundation for Statistical Computing), and SPSS 13.0 (SPSS Inc.). A *p*-value of <0.05 was statistically significant.

## Results

### Patient Characteristics

A total of 45 patients receiving operation for PDR were included. Another 90 propensity score-matched diabetic patients without PDR according to age, gender, and history of diabetes were enrolled as control group. Patient characteristics are shown in Table 1. There was no significant difference between the two groups in body mass index (BMI), diabetic complications, metabolic comorbidities, therapeutic strategies, and laboratory data.

**TABLE 1:** Patient characteristics.

	PDR (*n* = 45)	NDR (*n* = 90)	*p*-value
Age (year)	59.9 ± 11.3	60.9 ± 9.9	0.599
Male (*n*)	25	50	1.000
BMI (kg/m^2^)	24.4 ± 2.7	24.9 ± 3.8	0.400
History of diabetes (year)	10.0 (2.5, 16.7)	10.0 (2.0, 15.3)	0.277
**Complications (*n*)**			
Macrovascular	10	18	0.764
Kidney	7	13	0.864
Neuropathy	9	19	0.881
**Comorbidities (*n*)**			
Hypertension	17	29	0.521
Fatty liver	10	24	0.575
Dyslipidemia	12	19	0.469
**Medication (*n*)**			
Oral antidiabetic drugs	30	68	0.275
Metformin	22	48	0.626
Glycosidase inhibitors	12	22	0.779
DPP4 inhibitors	9	18	1.000
Insulin secretagogues	9	15	0.633
Insulin injection	24	43	0.543
Proton pump inhibitor	0	3	
**Laboratory data**			
Fasting glucose (mmol/L)	6.6 ± 2.3	6.6 ± 2.9	0.992
HOMA-IR	2.6 (1.8, 4.7)	2.5 (1.5, 4.6)	0.244
HbA1c (%)	9.6 ± 2.2	8.8 ± 2.3	0.144
Triglyceride (mmol/L)	1.40 (0.99, 2.06)	1.44 (1.02, 2.18)	0.590
Cholesterol (mmol/L)	4.37 ± 1.23	4.35 ± 1.11	0.929
Creatinine (μmol/L)	66.0 ± 30.4	69.9 ± 23.6	0.569

*PDR, proliferative diabetic retinopathy; NDR, diabetic patients without retinopathy; BMI, body mass index; DPP4, dipeptidyl peptidase-4; HOMA-IR, homeostasis model assessment of insulin resistance.*

### Reduced Gut Microbiota Diversity in PDR

**FIGURE 1:**
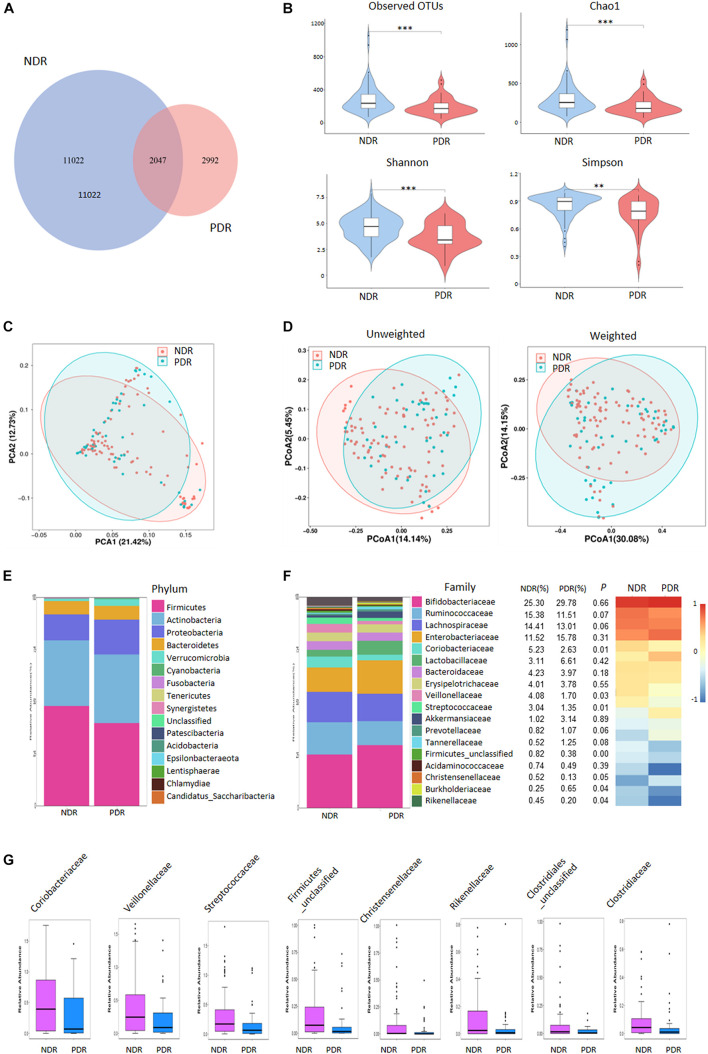
Gut microbiome diversity and structure comparison. **(A)** The OTUs in the two groups. **(B)** Alpha diversity comparison between the two groups. **(C)** PCA analysis based on the relative abundance of operational taxonomic units. **(D)** Unweighted/weighted Unifrac the score PCoA plot. **(E)** Component proportion of bacteria phylum in the two groups. **(F)** The comparison of bacteria at family level. **(G)** TOP8-altered bacteria at family level. ^∗∗∗^*p* < 0.001; ^∗∗^*p* < 0.01; ^∗^*p* < 0.05; PDR, proliferative diabetic retinopathy; NDR, diabetic patients without retinopathy; OTUs, operational taxonomic units; PCA, principal component analysis; PCoA, plot of principal coordinate analysis.

We performed 16S rRNA gene sequencing to assess the landscape of gut microbiome. A total of 8,569,316 high-quality valid tags (range: 46,269–80,826 per sample) were obtained. PDR group showed a decreased value of observed OTUs than NDR group, indicating reduced richness and bacterial diversity (Figure 1A). We further evaluated the differences in gut microbiome diversity between the two groups using alpha diversity and beta diversity. There was significantly lower alpha diversity of gut microbiome in the PDR group than that in the NDR group according to four independent indices including species richness indices (observed OTUs and Chao1) and species diversity indices (Shannon and Simpson) (Figure 1B). PCA displayed segregation of the microbiota between the two groups (Figure 1C). The score plot of principal coordinate analysis based on unweighted/weighted UniFrac distances showed differences in the structure and composition of bacterial community between the two groups (Figure 1D).

### Alterations of Gut Microbiome Signatures in PDR

To investigate the gut microbiome signatures, we assessed the relative abundance of gut bacterial composition by microbial taxon assignment at both phylum and family levels. At the phylum level, *Firmicutes*, *Actinobacteria*, and *Proteobacteria* accounted for most microbial communities (Figure 1E). However, no significant difference was found between the two groups including the *Firmicutes*/*Bacteroidetes* ratio. At the family level, PDR group showed significantly less abundant in 22 families including *Coriobacteriaceae*, *Veillonellaceae*, and *Streptococcaceae* and more abundant in two families (*Burkholderiaceae* and *Burkholderiales_unclassified*) than NDR group (Figure 1F). The representative high-abundance families are shown in Figure 1G. At the genus levels, there were 66 significantly different taxa between the two groups (Supplementary Figure 1).

**FIGURE 2:**
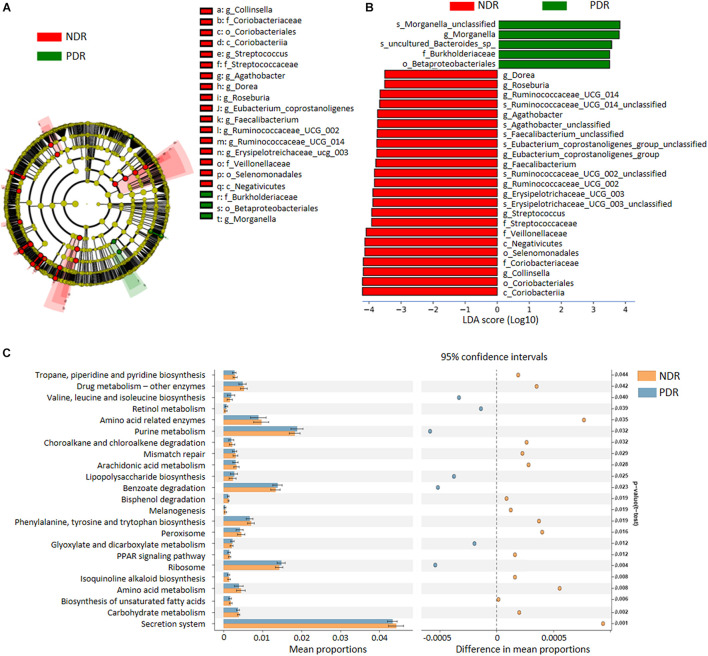
The discriminative operational taxonomic units and the predicted function. **(A)** The phylogenetic distribution of gut microbiota correlated with the two groups as shown by cladogram using LDA effect size analysis. **(B)** The differences of gut microbiota in abundance between the two groups (LDA > 3.5). **(C)** KEGG pathways predicted by PICRUSt analysis. PDR, proliferative diabetic retinopathy; NDR, diabetic patients without retinopathy; LDA, linear discriminant analysis; PICRUSt, phylogenetic investigation of communities by reconstruction of unobserved states.

To characterize the distinct microbial compositions, we further identified key discriminative OTUs in the PDR group relative to the NDR group using LEfSe analysis. There were 28 discriminative features with LDA scores > 3.5 level (Figure 2A). At the family level, PDR group was enriched by *Burkholderiaceae*, whereas NDR group was enriched by *Coriobacteriaceae*, *Streptococcaceae*, and *Veillonellaceae* (Figure 2B). At the genus level, the PDR group was enriched by *Morganella*, while the NDR group was enriched by *Collinsella*, *Streptococcus*, *Erysipelotrichaceae_UCG_003*, *Ruminococcaceae_UCG_002*, *Ruminococcaceae_UCG_014*, *Faecalibacterium*, *Eubacterium_coprostanoligenes_group*, *Agathobacter*, *Roseburia*, and *Dorea*.

To predict the functional alteration of gut microbiota in PDR patients, we performed PICRUSt analysis. The PDR group showed significant difference in nucleotide metabolism, amino acid metabolism, and lipid metabolism as compared with the NDR group (Supplementary Figure 2). There were 32 KEGG pathways that were significantly differentially enriched between the two groups (all *p* < 0.05) including secretion system, ribosome, peroxisome, purine metabolism, and carbohydrate metabolism (Figure 2C).

### Fecal Metabolic Profiles of PDR

**FIGURE 3:**
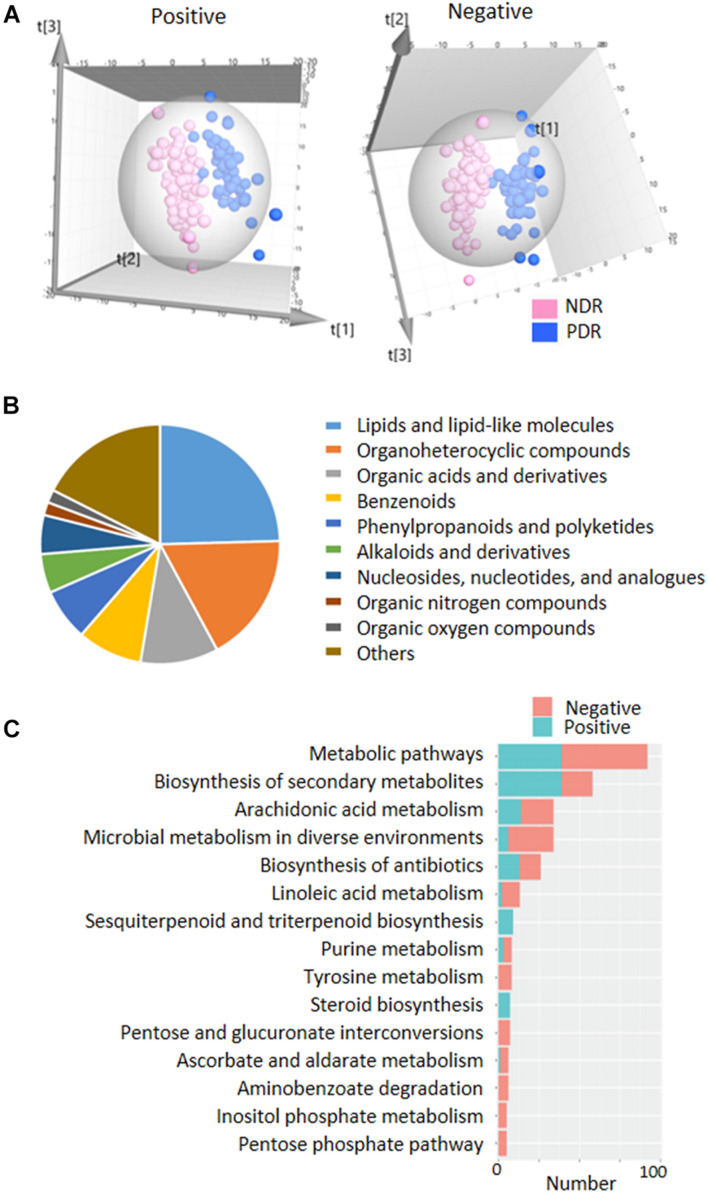
| Fecal metabolic profiles in patients with and without proliferative diabetic retinopathy. **(A)** The three-dimensional PLS-DA score plots of the two groups. **(B)** The classification of significantly altered metabolic features. **(C)** The enriched KEGG pathways of altered metabolic features. PDR, proliferative diabetic retinopathy; NDR, diabetic patients without retinopathy; PCA, principal component analysis; PLS-DA, partial least squares discriminate analysis.

To directly evaluate the influence of PDR on gut microbial metabolism, we performed UPLC-MS-based analysis of metabolomics using fecal samples. PCA score plots displayed that the QC samples in both ESI+ and ESI− models were clustered tightly together, indicating the analysis was stable and repeatable (Supplementary Figure 3). The partial least squares discriminate analysis (PLS-DA) score plots showed apparent separation between the PDR group and NDR group in both ESI+ and ESI− models (Figure 3A). There were 133 and 129 features in the ESI + and ESI− models significantly altered between the two groups according to selection criteria of *p* < 0.05, the variable importance in the projection (VIP) > 1, and | log2(fold change)| > 1. Most of the metabolites belonged to lipids and lipid-like molecules, organoheterocyclic compounds, organic acids and derivatives, and benzenoid superclasses (Figure 3B). They were significantly enriched in metabolic pathways including arachidonic acid metabolism, microbial metabolism in diverse environments, linoleic acid metabolism, and purine metabolism (Figure 3C). Of interest, 34 metabolic features were significantly enriched in microbial metabolism in diverse environment, indicating a potential impact of gut microbiota on the metabolic profiling.

### Cross-Omics Correlation Network Analysis

**FIGURE 4:**
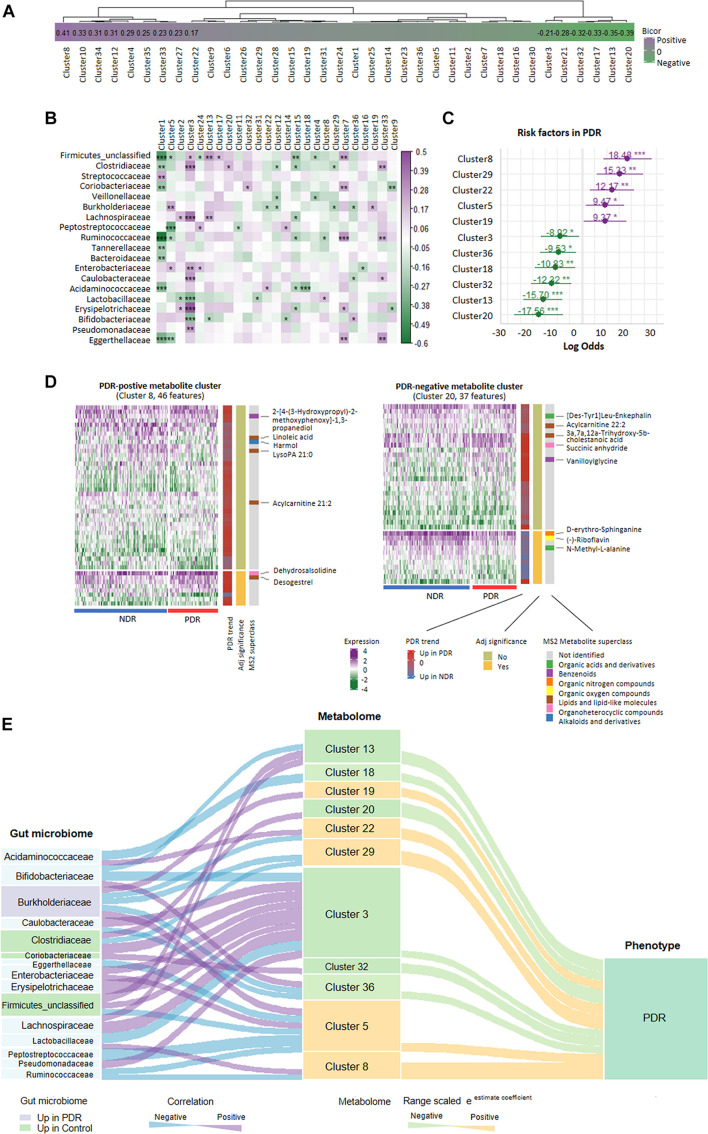
Relationship network between proliferative diabetic retinopathy-linked microbes and metabolites. **(A)** PDR-associated coabundance clusters as revealed by biweight midcorrelation analysis. **(B)** Significantly correlated gut microbiota families with coabundance clusters as shown by Spearman correlation analysis (***p < 0.001; **p < 0.01; *p < 0.05). **(C)** The positive/negative metabotypes of PDR as identified by logistic regression analysis. **(D)** The detailed description of representative positive/negative metabotypes of PDR as analyzed by coabundance correlation network. **(E)** Visualization of the gut microbiota-metabolites network according to Spearman correlation analysis (curve on the left) and logistic regression (curve on the right). PDR, proliferative diabetic retinopathy; NDR, diabetic patients without retinopathy.

Gut bacteria provide bioactive metabolites, which enter the bloodstream of host and affect the disease progression. We performed cross-omics analysis to show the link between gut bacteria, the associated metabolites, and PDR. A total of 8,208 identified MS1/MS2 features were filtered by top 75% median absolute deviation from 10,944 features. First, we classified the metabolites into 36 coabundance clusters including 15 clusters that were significantly correlated with PDR (*p* < 0.05, Figure 4A). Various kinds of mechanism can come up with clusters of coabundance metabolites such as: (1) metabolites correlated with the same biological process/pathway; (2) the same/similar precursor metabolite chemical modification; and (3) metabolites coyielded by the specific gut microbiome. Second, we performed correlation analysis between gut microbiota families and the metabolite clusters. We found a significant correlation between 19 families and 25 clusters (Figure 4B). For instance, *Burkholderiaceae* was significantly positively correlated with clusters 5 and 19 but negatively correlated with clusters 12, 22, 29, and 36, while *Coriobacteriaceae* was significantly positively correlated with clusters 7 and 32. We further entered the gut microbiota-correlated 25 metabolite clusters into logistic regression and identified 11 clusters that significantly contribute to the risk of PDR (Figure 4C). The detailed information of the 11 clusters including MS2 metabolites, MS2 superclass, and compassion significance between the PDR group and NDR group are provided in Supplementary Table 1. According to the contribution power (risk ratio), we classified the 11 clusters into five PDR-positive metabotypes and six PDR-negative metabotypes. The most representative risk (cluster 8) and protective metabotypes (cluster 20) for PDR are shown in Figure 4D. Cluster 8 contained 46 features including desogestrel and dehydrosalsolidine that were significantly more abundant in the PDR group, whereas cluster 20 contained 37 features including N-methyl-L-alanine and D-erythro-sphinganine that were significantly less abundant in the PDR group as compared with the NDR group. Finally, we construct an interaction network for 15 gut microbiota families and the 11 PDR-positive/negative metabotypes, suggesting that gut microbiota might contribute to the development of PDR through interacting with metabolites (Figure 4E).

### Predictive Model for PDR

**FIGURE 5:**
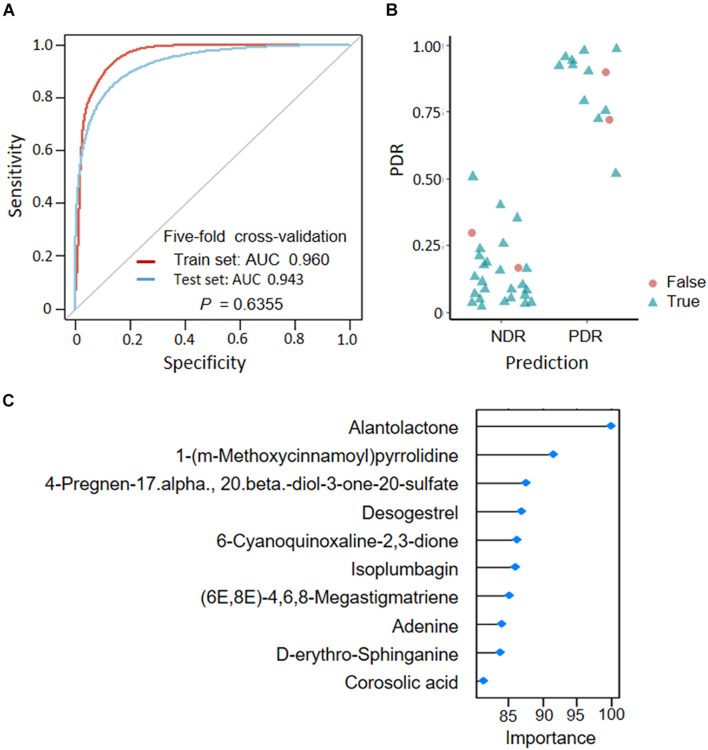
Predicting proliferative diabetic retinopathy based on fecal metabolites. **(A)** Classification performance as shown by AUC. **(B)** Predicted probabilities for each sample. **(C)** TOP10 strongest discriminant metabolites in the classifier. PDR, proliferative diabetic retinopathy; NDR, diabetic patients without retinopathy; AUC, area under curves.

To assess the predictive role of fecal metabolites in PDR, we trained SVM classifiers on patient fecal metabolite profiles. We divided all patients into a train set and a test set according to a ratio of 7:3. Classification performance was evaluated by AUC. We found that the MS2 metabolites could well distinguish PDR patients from NDR patients, as shown by AUCs of 0.960 and 0.943 in train and test sets (Figure 5A). The metabolites model presented high accuracy in predicting PDR with sensitivity of 0.846 and specificity of 0.936 (Figure 5B). The most discriminate metabolites included alantolactone, desogestrel, adenine, D-erythro-sphinganine, and corosolic acid (Figure 5C).

## Discussion

Gut-retina axis has been proposed according to the mice experiments (Floyd and Grant, 2020) and hypothesize that diabetic-associated microbiome could lead to increased inflammation and vascular permeability, which influence the development and progression of DR (Fernandes et al., 2019). However, alterations of gut microbiota have not been directly linked to DR in human studies. A previous study using fecal colony culture and PCR strategy did not find a significant difference in the abundance of *Bacteroides* between diabetic patients with and without DR (Moubayed et al., 2019). A pilot-scale study using only 16S rRNA sequencing showed dysbiosis in microbiomes in 28 DR patients as compared with 30 healthy individuals and 24 type 2 diabetic patients (Das et al., 2021). This is a pioneering study that explores the DR-associated alterations of human gut microbiome and metabolome. We enrolled PDR patients as the DR group, which were compared with the NDR group, to maximize the diversity between DR and NDR. We demonstrated sharply decreased bacteria abundance and gut microbiota diversity in diabetic patients with PDR as compared with those without DR. The loss of microbial taxonomic diversity is frequently observed in many human diseases such as diabetes and cancer and is recognized to be associated with systemic inflammation (Estaki et al., 2016; Arnoriaga-Rodriguez and Fernandez-Real, 2019), which play a clear role in the pathogenesis of DR (Duh et al., 2017). Therefore, we suppose that microbial diversity might reflect the severity of DR.

Moreover, we showed the change of microbiota composition and specific population of bacterial species in diabetic patients with PDR as compared with those without DR. There was no significant difference in bacterial abundance at phylum level between the two groups. However, at family level, PDR was found to be associated with significantly decreased abundance of a series of bacteria including *Coriobacteriaceae*, *Veillonellaceae*, and *Streptococcaceae*. Several bacteria have known function particularly in metabolic diseases. For instance, *Coriobacteriaceae* may regulate host glucose homeostasis *via* liver energy metabolism and protect against hyperglycemia (Liu et al., 2018). *Veillonellaceae* are the key organisms in human gut that metabolize lactate (Scheiman et al., 2019), thereby reducing the risk of developing diabetic complication including PDR (Mieno et al., 2019). In addition, *Clostridiales_unclassified*, *Ruminococcaceae*, *Firmicutes_unclassified*, *Clostridiaceae*, and *Rikenellaceae* were the top bacterial taxa at the family level contributing to the ClpB-like gene function that leads to reduced fat mass (Arnoriaga-Rodriguez et al., 2020). In contrast to the bacteria with decreased abundance, the family *Burkholderiaceae* was the only bacterial taxa that are enriched in the gut of PDR patients and the key discriminative microbial marker as identified by LEfSe analysis. *Burkholderiaceae* is a known heterotrophic bacteria that was reported to colonize in the gut of patients with immunosuppression (Yang et al., 2016) and positively correlate with chemokine IP-10, inducing systemic inflammation (Zhang et al., 2019). Moreover, *Burkholderiaceae*, along with *Coriobacteriaceae* and *Streptococcaceae*, were closely correlated with altered glutamate metabolism (Palomo-Buitrago et al., 2019), which has been proven to be an early pathogenic event in the development of DR (Lieth et al., 1998).

We further demonstrated that not only the abundance and composition of gut microbiome but also the gut-derived metabolites displayed PDR-specific biosignature. The significant differentially expressed metabolites were enriched in metabolic pathways, such as, linoleic acid metabolism, purine metabolism, tyrosine metabolism, and carbohydrate metabolism. Some metabolic pathways including arachidonic acid metabolism and purine metabolism were predicted to be altered by microbiome and further proved by metabolome. We found that the arachidonic acid metabolites such as hydroxyeicosatetraenoic acids (HETEs) and leukotriene, which are known mediators for DR development (Bapputty et al., 2019; Wang et al., 2020), were increased in the fecal of PDR patients, making them as the potential diagnostic markers and therapeutic targets. Our results were consistent with previous studies that HETEs were also increased in the serum of DR patients as compared with NDR patients (Xuan et al., 2020). On the other hand, there were 34 fecal metabolites including vanillate, D-galactonate, D-gluconic acid, and aerobactin that were significantly enriched in microbial metabolism, showing an extensive interplay between the gut microbiota and the host through metabolic exchange and substrate cometabolism (Nicholson et al., 2012).

We next revealed an integrated cross-omics framework to better understand the link between gut microbiome and metabolome particularly under the circumstance of PDR. We enriched coexpression-based clusters to classify metabolites with similar physicochemical properties. The method greatly shortened the numbers of metabolomics parameters from thousands of metabolite features to dozens of metabolite clusters, which made the risk analysis using logistic regression available. We then identified several metabolite clusters that were significantly associated with PDR risk. For instance, the metabolic pattern of cluster 8, including 33 upregulated (e.g., desogestrel and acylcarnitine 21:2) and 13 downregulated metabolite features (e.g., LysoPA 21:0 and linoleic acid) in PDR patients as compared with NDR patients, sharply increased the risk of PDR by 18.5-fold in diabetic patients. In contrast, the metabolic pattern of cluster 20, including 12 upregulated (e.g., succinic anhydride) and 25 downregulated metabolite features (e.g., acylcarnitine 22:2 and (−)-riboflavin) in PDR patients, dramatically reduced the PDR risk by 17.6-fold. The results not only confirmed the previously identified PDR-associated metabolites such as the arginine and carnitine metabolites (Liew et al., 2017; Sumarriva et al., 2019) but also provided new candidates from unlabeled metabolites. Furthermore, we correlated the gut microbiota with the metabolic phenotype and found significant relationships between certain bacteria families and PDR-associated metabolite clusters. For example, cluster 3, the most microbial-affected metabolite cluster containing 19 organic acids and derivatives, was significantly correlated with nine microbial families including *Clostridiaceae*, *Lachnospiracea*, *Lactobacillaceae*, and *Bifidobacteriaceae*. The PDR-negative metabotype cluster 32 was only positively correlated with *Coriobacteriaceae*. The results shed light on the PDR-linked microbe-metabolite interaction.

There were limitations in this study. First, the sample size was relatively small. Therefore, we used propensity score-matched cohort to minimize the selection bias. Second, there were some confounding factors. Although the diabetic complications and medications, which have impact on the gut microbiome and metabolomics, did not differ significantly between the two groups, the diet habit and lifestyle were not controlled and may also affect the results. Third, the casual relationship between gut microbe-metabolite and PDR could not been determined by this study. Lastly, most coabundance metabolite clusters remained largely uncharacterized and need further exploration. The therapeutic significance of restoration of gut microbiota in PDR also needs to be proved.

In conclusion, to the best of our knowledge, it is the first study to identify the alterations in both gut microbial community and metabolism in diabetic patients with PDR versus NDR. There was sharply reduced richness and bacterial diversity in PDR patients, making supplemental probiotics a potential therapeutic strategy through restoring the balance. Moreover, there was a specific interplay between gut microbiome and host metabolome under the circumstances of PDR. A fecal metabolite-derived classifier was constructed and could effectively discriminate PDR patients from NDR patients. Our results not only help to better understand the physiopathological mechanisms of PDR but also provide novel strategies in disease prevention, diagnosis, and treatment.

## Data Availability Statement

The data are deposited in the SRA repository, accession number is PRJNA753622 (Bioproject ID).

## Ethics Statement

The studies involving human participants were reviewed and approved by Ethical Committee of the Second Affiliated Hospital, Zhejiang University School of Medicine (approval number 2020776). The patients/participants provided their written informed consent to participate in this study.

## Author Contributions

PY designed the study, obtained the samples and clinical records, and wrote the manuscript. XZ analyzed the data. YX, JX, and XS obtained the samples and clinical records. KY supervised the study and revised the manuscript. All authors contributed to the article and approved the submitted version.

## Conflict of Interest

The authors declare that the research was conducted in the absence of any commercial or financial relationships that could be construed as a potential conflict of interest.

## Publisher’s Note

All claims expressed in this article are solely those of the authors and do not necessarily represent those of their affiliated organizations, or those of the publisher, the editors and the reviewers. Any product that may be evaluated in this article, or claim that may be made by its manufacturer, is not guaranteed or endorsed by the publisher.

## Footnotes

1
https://www.mzcloud.org/

2
http://www.chemspider.com/
