# Supplementary material for: Alterations of the Gut Microbiome and Metabolome in Patients With Proliferative Diabetic Retinopathy
Source: Front Microbiol. 2021 Sep 8;12:667632. doi: 10.3389/fmicb.2021.667632 (PMC8457552; doi:10.3389/fmicb.2021.667632)
Supplement: Supplementary file 1 [file Data_Sheet_1.docx]

Supplementary Material


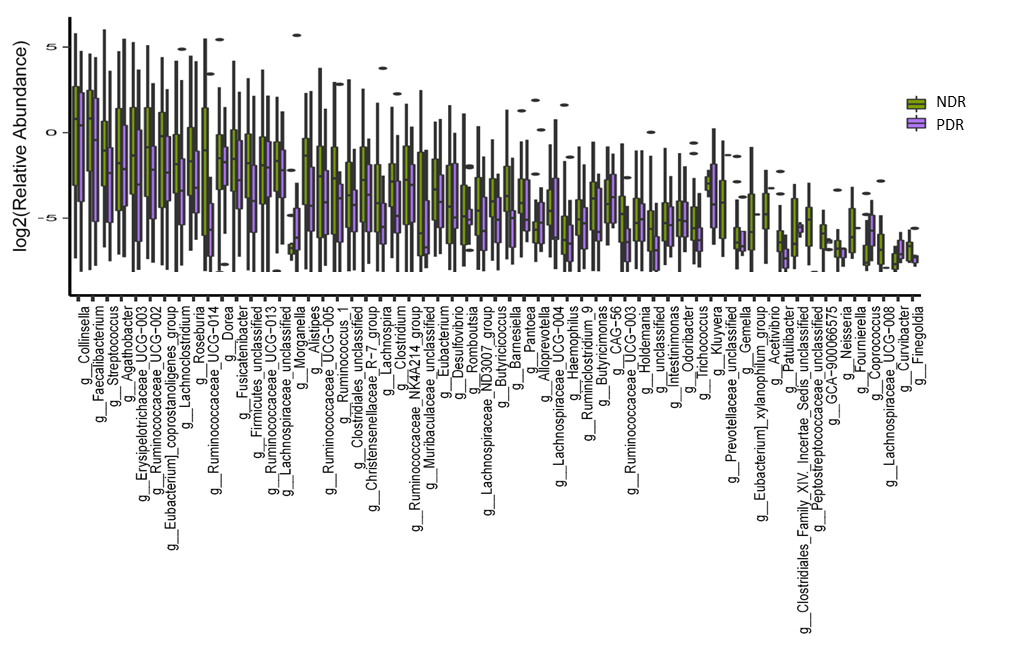


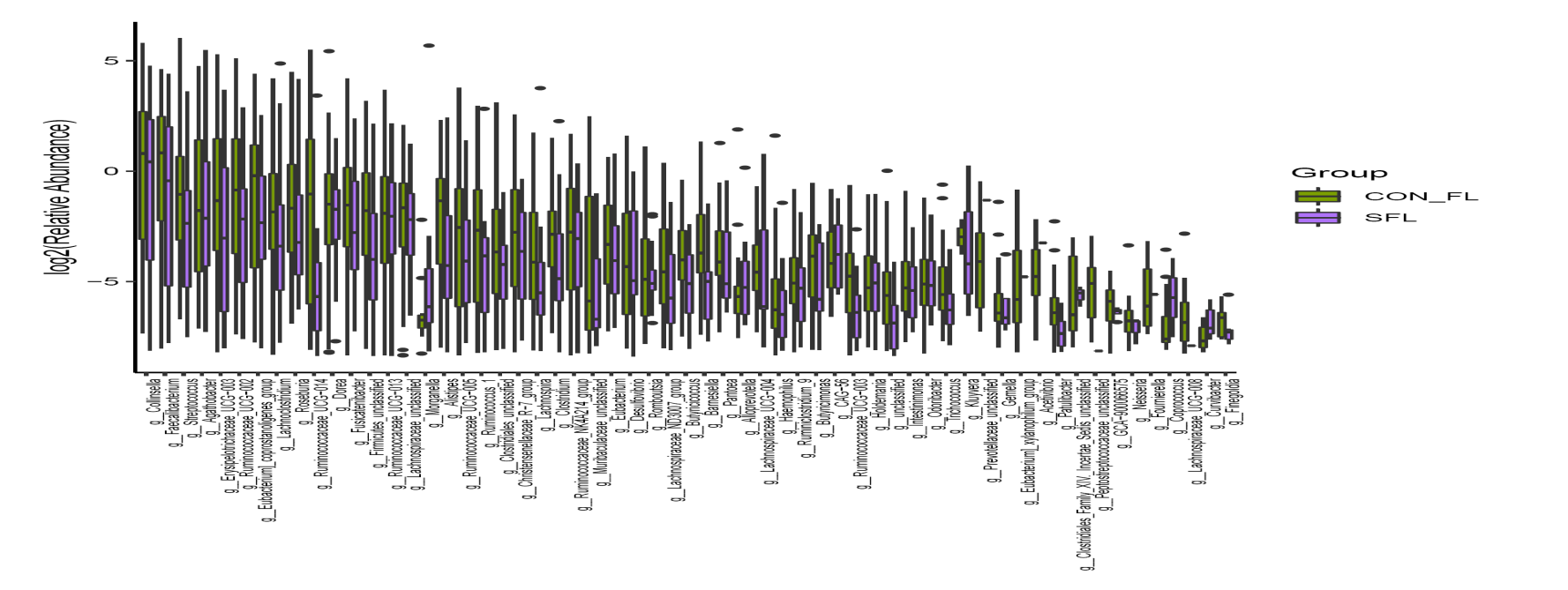

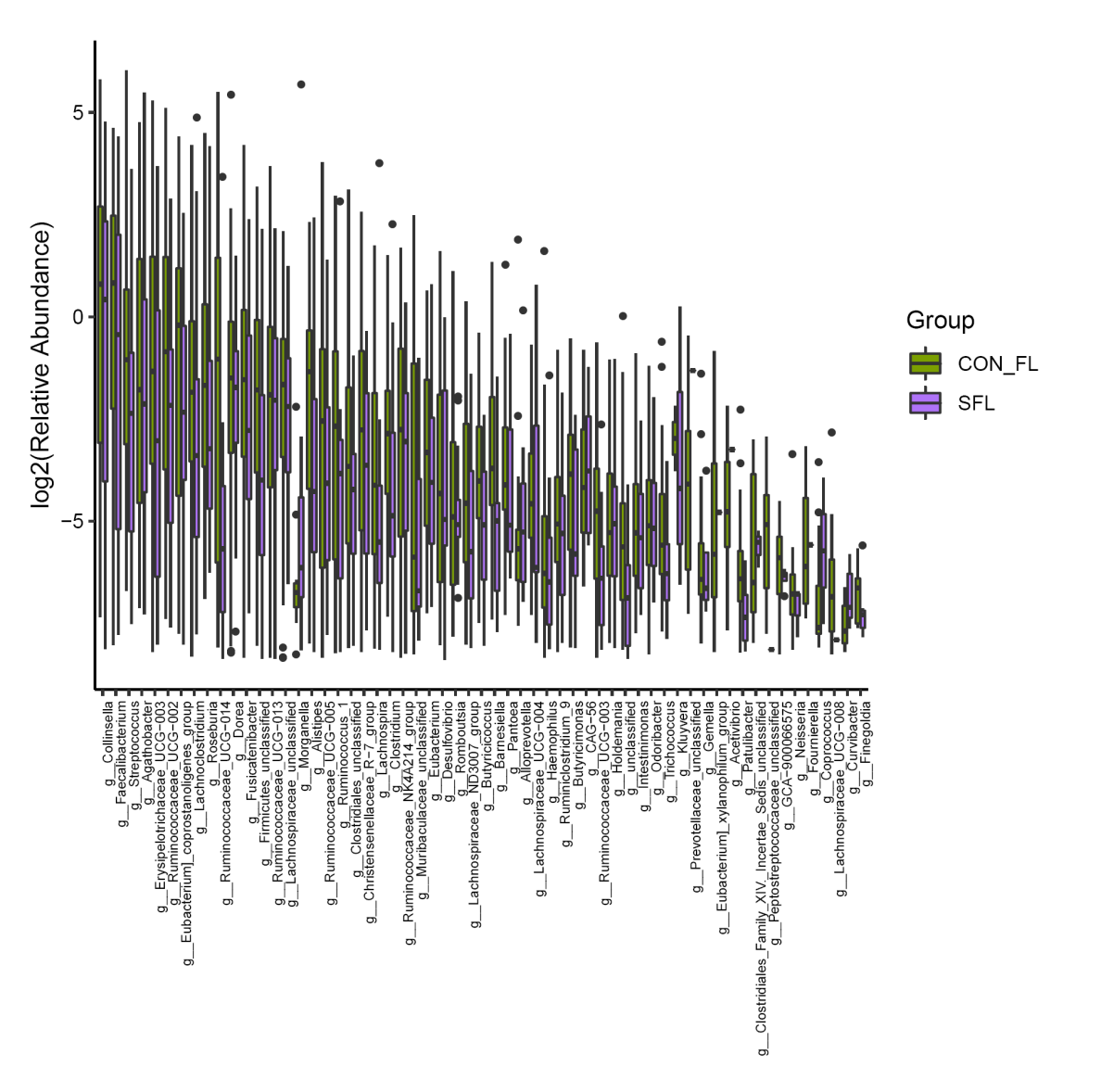

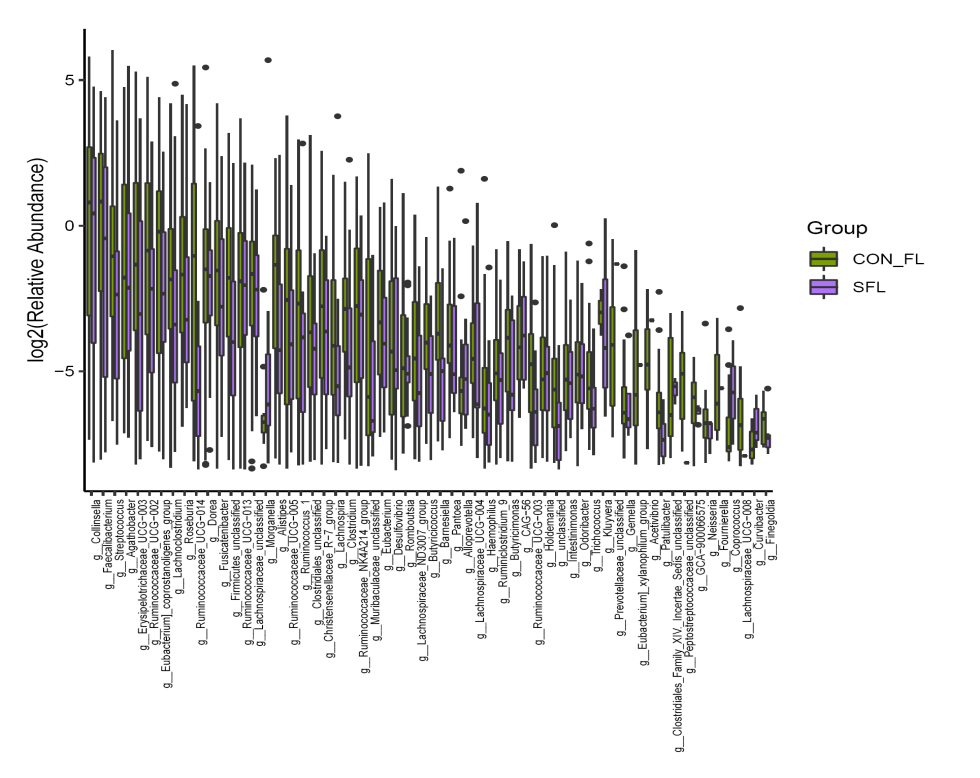

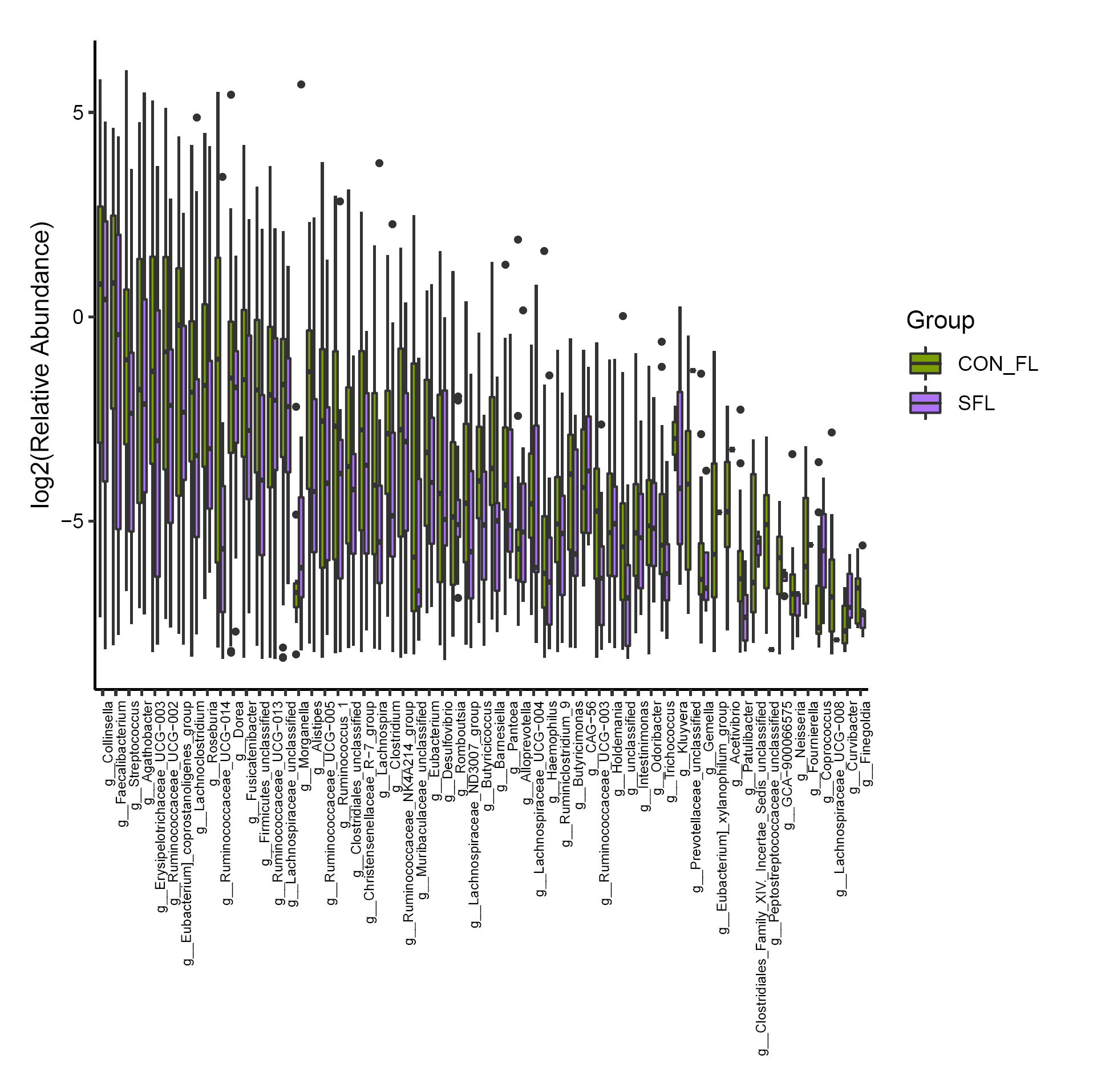


NDR

PDR

**Supplementary Figure 1.** The significant altered bacteria at genus level in proliferative diabetic retinopathy (PDR) patients .


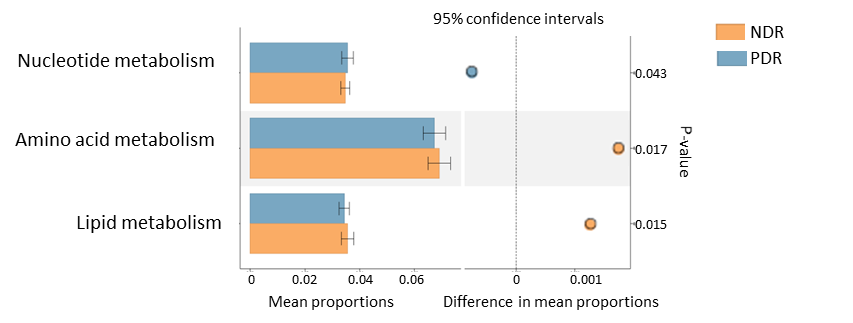


**Supplementary Figure 2.** The enriched pathways of significantly differential expressed metabolites between patients with and without proliferative diabetic retinopathy (PDR).


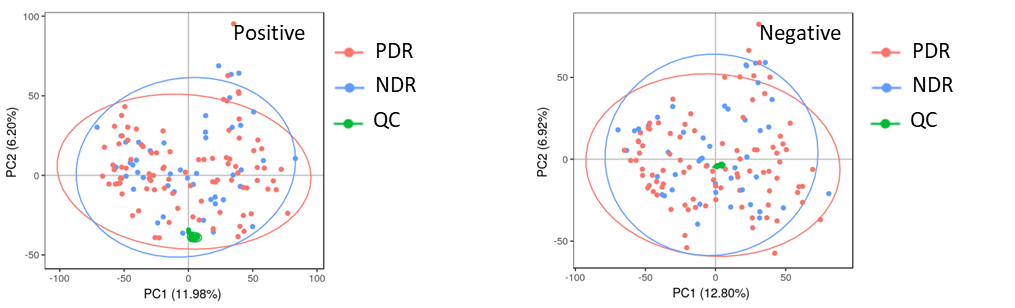


**Supplementary Figure 3.** Principal component analysis (PCA) score plots of metabolomics data. PDR, proliferative diabetic retinopathy; QC, quality control.
